# MYC-positive follicular lymphoma complicated by refractory lactate and clonal evolution: a case report

**DOI:** 10.3389/fonc.2026.1781821

**Published:** 2026-02-25

**Authors:** Yu-Qing Wang, Ke-Xin Wang, Hong-Juan Yu, Kun-Peng Yang, Dan-Yang Li, Yue Wang, Yue Liu, Jiao Meng, Shu-Ye Wang

**Affiliations:** 1 Department of Hematology, The First Affiliated Hospital of Harbin Medical University, Harbin, China; 2 Department of Pathology, The First Affiliated Hospital of Harbin Medical University, Harbin, China

**Keywords:** chemoresistance, clonal evolution, follicular lymphoma, lactic acidosis, metabolic reprogramming, MYC protein

## Abstract

Follicular lymphoma (FL) typically follows an indolent clinical course, however, a subset of patients develops an aggressive and treatment refractory phenotype. Here we report a fatal case of a 46-year-old male with FL grade 3A characterized by recurrent severe tumor-associated lactic acidosis (>15 mmol/L) and rapid therapeutic failure. The patient presented with B symptoms, extensive lymphadenopathy, profound hyperlactatemia, and serum IgM-κ monoclonal protein. Immunohistochemistry confirmed FL grade 3A diagnosis and revealed MYC protein overexpression within 4 months, manifesting new nodal lesions, markedly elevated lactate levels, sustained MYC overexpression, and emergence of both IgG-κ and IgM-κ monoclonal proteins, a serological signature of ongoing clonal evolution. Subsequent treatments (R-CHOP, R-CDOP, BR, and G-EPOCH) over 6 months failed to achieve durable disease control. The clinical course was dominated by refractory, recurrent type B lactic acidosis that correlated directly with tumor activity. The patient ultimately died of fulminant lactic acidosis complicated by tumor lysis syndrome, 7 months after initial diagnosis. This case identifies an ultra-aggressive variant of FL defined by MYC-driven metabolic dysregulation and dynamic clonal evolution, underscoring the need for early recognition and development of novel therapeutic strategies targeting metabolic reprogramming.

## 1 Introduction

Follicular lymphoma (FL) is the most common indolent B-cell non-Hodgkin lymphoma (1). Management strategies for patients with low tumor burden often include initial observation, which does not adversely impact overall survival (2). Nonetheless, a subset of patients exhibit early progression, histologic transformation, or aggressive metabolic complications that substantially worsen prognosis (3, 4). FL grade 3A, although classified within the FL spectrum, demonstrates higher proliferative activity and may behave more aggressively than lower grade counterparts (5).

Metabolic abnormalities, particularly lactic acidosis, represent rare but clinically significant complications in lymphoma (6). Type B lactic acidosis arises from excessive glycolytic activity of malignant cells and reflects profound metabolic reprogramming (7). While this phenomenon is well documented in aggressive lymphomas such as diffuse large B-cell lymphoma (DLBCL) (8) and Burkitt lymphoma (BL) (9), it is exceedingly uncommon in FL. The occurrence of lactic acidosis at presentation or in early-stage, untreated FL is even more exceptional.

Monoclonal immunoglobulins are occasionally observed in B-cell lymphomas, though they are more characteristically associated with lymphoplasmacytic lymphoma (10). The co-occurrence of monoclonal immunoglobulins with recurrent lactic acidosis in FL is highly atypical. This distinctive triad may signal underlying biological alterations, such as accelerated clonal evolution, metabolic transformation, or dysregulation of oncogenic pathways including MYC signaling (11).

Here, we report an exceptionally aggressive case of FL grade 3A complicated by recurrent, severe lactic acidosis and rapid progression despite multiple lines of therapy. Notably, the tumor cells demonstrated prominent MYC protein overexpression, providing a plausible molecular link between the observed metabolic derangement and the exceptionally aggressive clinical course. In addition, the patient initially presented with an IgM-κ monoclonal protein and later developed an additional IgG-κ component, suggesting ongoing clonal evolution that paralleled the metabolic escalation. The case illustrates a highly unusual metabolic phenotype in FL and underscores the importance of early recognition, metabolic monitoring, and exploration of alternative therapeutic approaches.

## 2 Case presentation

A 46-year-old male with no significant past medical history presented with a one-month history of a painless right inguinal mass accompanied by progressive fatigue, chest tightness, drenching night sweats, intermittent fever (maximum 38.6°C), and an unintentional 5kg weight loss. He denied alcohol abuse, diabetes, or use of medications associated with lactic acidosis.

Imaging studies revealed multiple enlarged lymph nodes in the left cervical (maximum diameter 2.1 cm), bilateral axillary (right 2.5 cm, left 2.3 cm), right inguinal (3.8 cm), and abdominal regions (retroperitoneal nodes up to 3.2 cm), along with splenomegaly (14.5 cm in length). Initial laboratory evaluation demonstrated leukocytosis (white blood cell count 10.09 × 10^9^/L, reference range: 3.5-9.5), lymphocytosis (4.2 × 10^9^/L, reference range: 1.1-3.2), anemia (hemoglobin 108g/L, reference range: 130-175), and thrombocytopenia (platelet count 50 × 10^9^/L, reference range: 125-350). Notably, serum lactate was markedly elevated (>15 mmol/L, reference range: 0.5-2.2), whereas lactate dehydrogenase (LDH) and β2-microglobulin levels remained within normal limits. ECOG performance status was 1.

A biopsy of the right inguinal lymph node revealed disrupted architecture with sheets of large atypical lymphoid cells exhibiting irregular nuclear contours, prominent nucleoli, and a sparse background of small lymphocytes. Pathological review was independently performed by three experienced hematopathologists (Y.-W.C., D.-Y.L., and Z.-F.G.) in accordance with the current World Health Organization classification criteria, and a consensus diagnosis was reached (12). Immunohistochemistry (IHC) showed positivity for CD10, CD20, BCL2, BCL6 (focal), MUM1, C-MYC, and PAX5, with a Ki-67 proliferation index of 70%. EBER was negative. Bone marrow (BM) aspiration demonstrated 28.5% lymphoma cell involvement. Flow cytometry identified a monoclonal B-cell population expressing CD19, CD20, CD22, CD38, FMC7, and kappa light chains. Cytogenetic analysis detected dup (1)(q11q21) and t(3;11)(q29;q21), no rearrangement of *MYC, BCL2*, or *BCL6* were identified, and the *MYD88*^L265P^ mutation was not detected. Serum immunofixation revealed an IgM-κ monoclonal component. These findings supported a diagnosis of FL, grade 3A, stage IVB, high risk (FLIPI-1: 3).

**Figure 1:**
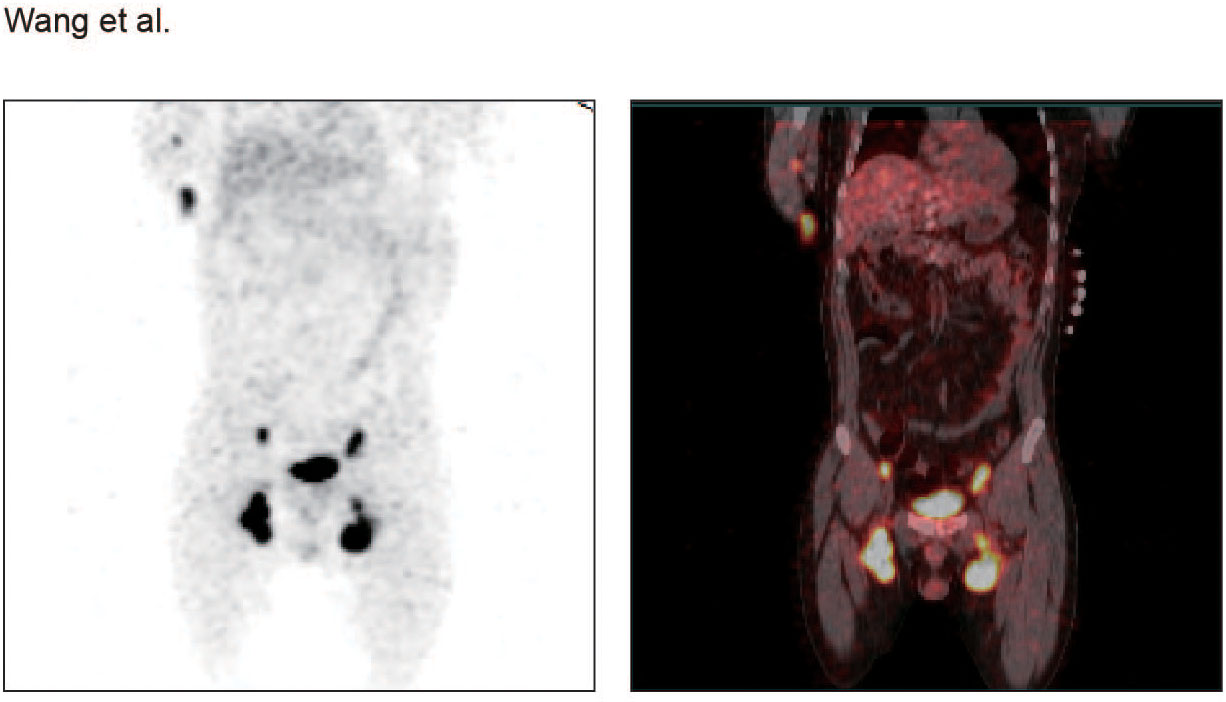
PET-CT imaging of the patient.

**Figure 2:**
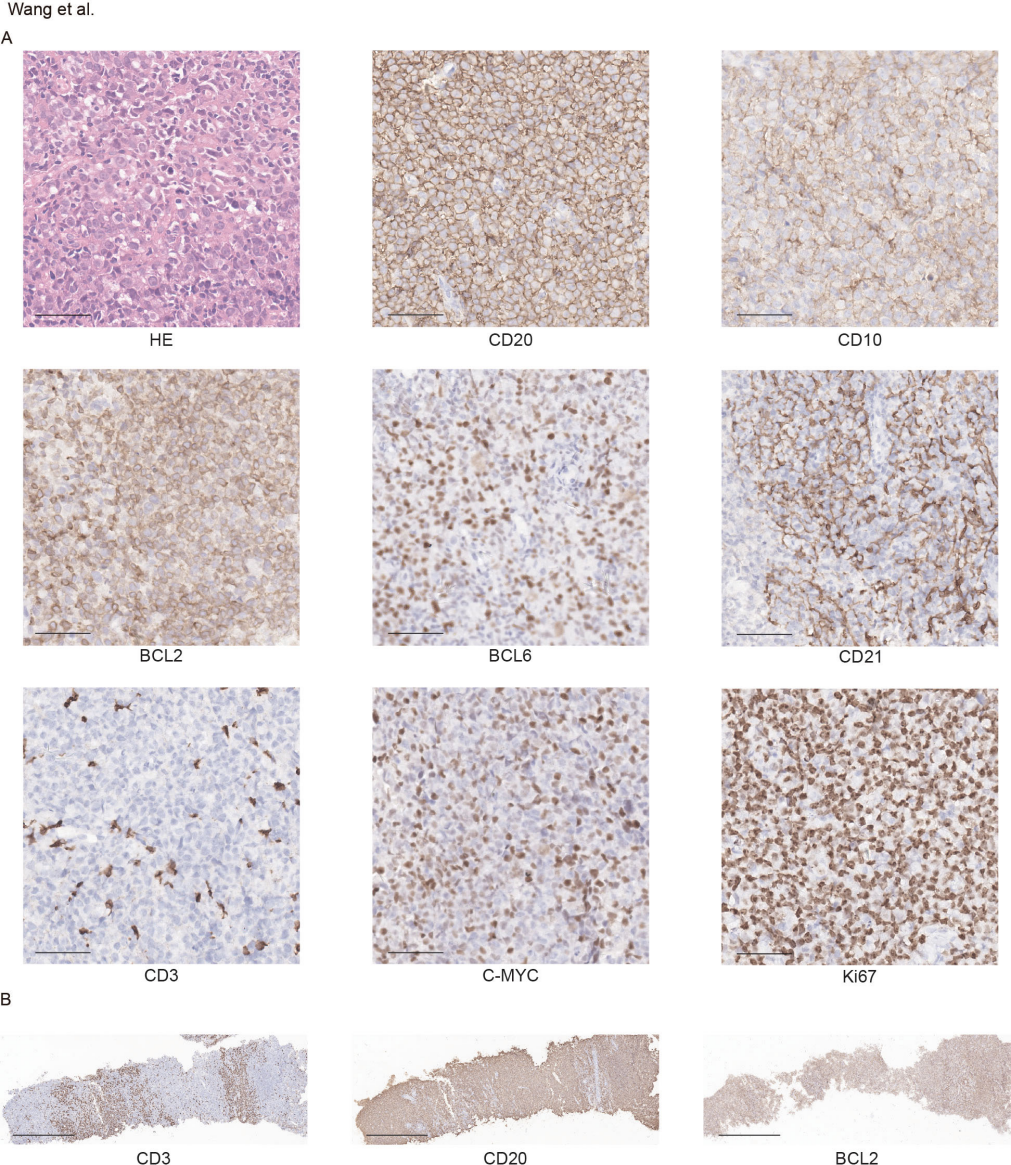
Diagnostic histopathological and immunohistochemical features of follicular lymphoma. **(A)** H&E and immunohistochemical staining for CD20, CD10, BCL2, BCL6, CD21, MYC, Ki67, and CD3 of follicular lymphoma. The ruler represents 50 μm. **(B)** Immunohistochemical staining for CD3, CD20, and BCL2. The ruler represents 250 μm.

**Figure 3:**
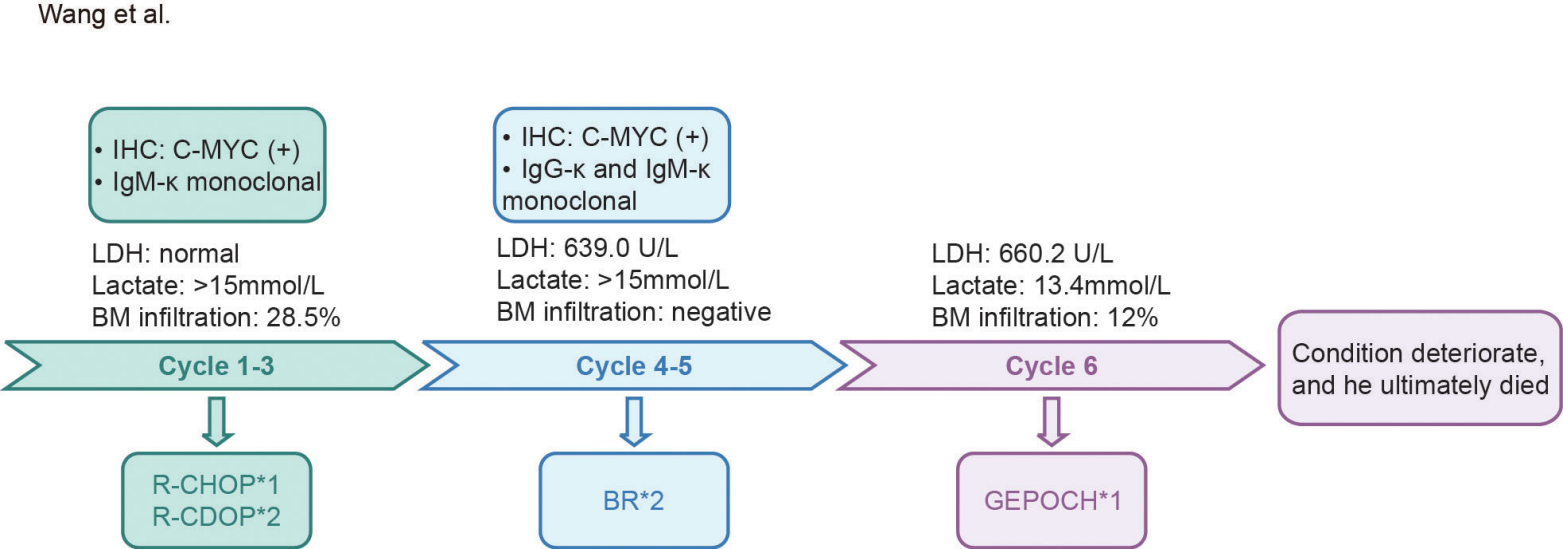
Diagnostic and therapeutic timeline of a patient with follicular lymphoma. Diagnostic and therapeutic timeline of a patient with follicular lymphoma.

The patient received one cycle of R-CHOP (rituximab 375 mg/m^2^, cyclophosphamide 750 mg/m^2^, doxorubicin 50 mg/m^2^, vincristine 1.4 mg/m^2^, and prednisone mg/m^2^ days 1-5), leading to improvement in lactic acidosis (lactate decreased to normal) and reduction of BM infiltration to 6%. Due to cardiotoxicity concerns, the regimen was switched to R-CDOP (liposomal doxorubicin replacing conventional doxorubicin). After three cycles, bone marrow minimal residual disease became negative by flow cytometry, and previously enlarged lymph nodes regressed by more than 50%. However, PET-CT revealed a newly enlarged left inguinal lymph node (3.2 cm) (**Figure 1**), and serum lactate markedly re-elevated to over 15 mmol/L. Repeat biopsy confirmed persistent FL grade 3A with a high Ki67 index (70%), and increased MYC protein expression (50.1%) (**Figure 2A**), in the absence of *MYC*, *BCL2*, and *BCL6* rearrangements. Morphologically, the neoplastic follicles were composed of a mixture of centrocytes and increased numbers of centroblasts, without diffuse sheets of exclusively large cells. CD21 immunostaining demonstrated partially preserved follicular dendritic cell meshworks, while CD3 staining highlighted residual interfollicular T lymphocytes interspersed between tumor cell aggregates (**Figures 2A, B**). In addition, targeted sequencing revealed genetic abnormalities, including *ARID1A, CCND3, ID3, LRP1B, MYD88* (non-L265P), and *NOTCH2* mutations (**Table 1**). Serum immunofixation now detected both IgG-κ and IgM-κ monoclonal components, indicating clonal evolution. LDH increased to 639.0 U/L (reference range 120–250 U/L). The patient subsequently received two cycles of BR (bendamustine 90 mg/m^2^ days 1-2, rituximab 375 mg/m^2^ day 1). Despite treatment, disease continued to progress, manifesting as generalized lymphadenopathy and an increase in BM infiltration to 12%. Serum lactate again rose to 13.4mmol/L, and LDH increased markedly to 660.2 U/L. One cycle of G-EPOCH (obinutuzumab 1000mg days 1,8,15; etoposide 50 mg/m^2^ days 1-5; cyclophosphamide 750 mg/m^2^ day 5; doxorubicin 10 mg/m^2^/day continuous infusion days 1-4; vincristine 0.4 mg/m^2^/day continuous infusion days 1-4; prednisone 60 mg/m^2^/day days 1-5) produced a transient reduction in serum lactate to 2.1mmol/L. However, prior to the planned second G-EPOCH cycle, both serum lactate and white blood cell (WBC) counts rapidly increased, with WBC rising to 32.7 × 10^9^/L, predominantly neutrophils with pronounced left shift, peripheral blood smear showed no circulating lymphoma cells. Concurrent bone marrow examination revealed persistent lymphoma infiltration at 13%. Serum lactate exceeded 15mmol/L and LDH increased to 2221.9 U/L. The patient subsequently developed recurrent severe hyperlactatemia, acute hepatic and renal dysfunction, and rapidly worsening lactic acidosis complicated by tumor lysis syndrome. Despite intensive supportive treatment including continuous sodium bicarbonate infusion, and aggressive hydration, his condition continued to deteriorate. The patient died 7 months after initial diagnosis due to multi-organ failure secondary to refractory lactic acidosis and tumor lysis syndrome (**Figure 3**).

**Table 1:** Pathogenic gene mutations identified by next-generation sequencing.

Gene (symbol)	Transcript ID	Exon	Nucleotide change	Protein change	Variant class	VAF (%)
ARID1A	NM_006015.4	Exon 1	c.1060C>T	p.Gln354	Nonsense (Grade 2)	6.24
CCND3	NM_001760.4	Exon 5	c.847A>G	p.Thr283Ala	Missense (Grade 2)	6.98
ID3	NM_002167.4	Exon 1	c.113_114insTAGCTGAGGAGCCGCT	p.Leu40fs	Frameshift Insertion (Grade 2)	3.38
LRP1B	NM_018557.2	Exon 44	c.7328G>A	p.Arg2443His	Missense (Grade 2)	5.25
MYD88	NM_002468.4	Exon 4	c.728G>A	p.Ser243Asn	Missense (Grade 2)	7.58
NOTCH2	NM_024408.3	Exon 8	c.1274A>G	p.Asn425Ser	Missense (Grade 3)	49.21

## 3 Discussion

This report presents an exceptionally aggressive and ultimately fatal case of FL grade 3A, characterized by two rare and life-threatening features, recurrent severe type B lactic acidosis and the sequential emergence of IgM-κ and IgG-κ monoclonal proteins. These profound clinical manifestations were associated with persistent MYC protein overexpression, suggesting a distinct, metabolically driven, high-risk phenotype within the FL spectrum.

While FL grade 3A typically exhibits more proliferative biology compared with lower-grade disease, its clinical course is generally indolent to intermediate and does not usually resemble the fulminant trajectory seen in transformed or high-grade lymphomas (13). Most patients with FL grade 3A achieve initial responses to standard immunochemotherapy, with median overall survival exceeding 10 years (5). Thus, the extreme aggressiveness of this case, with progression within 4 months of initial therapy and death within 7 months, is highly atypical and implies the presence of additional biological drivers beyond histologic grade alone.

In this patient, the rapid evolution of clinical symptoms closely paralleled profound molecular and immunophenotypic changes, reflecting escalating biological complexity. The sequential appearance of distinct monoclonal immunoglobulin components indicates ongoing clonal evolution and aberrant B-cell differentiation. This phenomenon, while occasionally seen in lymphoplasmacytic lymphoma, is exceptionally rare in FL and suggests dynamic clonal diversification. The emergence of dual immunoglobulin isotypes may reflect either the expansion of distinct subclones or aberrant class-switch recombination within a single evolving clone. Thus, this immunophenotypic plasticity likely contributed to therapeutic resistance and disease progression.

Remarkably, this immunophenotypic aberration coincided with persistent MYC overexpression, which may represent a critical link between clonal instability and the catastrophic metabolic phenotype observed. In this case, 50.1% of tumor cells demonstrated positive MYC nuclear staining, a level that has been associated with adverse prognosis in aggressive B-cell lymphomas independent of MYC rearrangement status (14). As a master transcriptional regulator, MYC disrupts normal B-cell maturation and reprograms cellular metabolism (15–17). Compared with normal cells, malignant B cells with MYC dysregulation exhibit enhanced aerobic glycolytic, preferentially converting pyruvate to lactate even in the presence of oxygen, a hallmark of the “Warburg effect” (18). MYC, often in cooperation with HIF-1α, directly regulates key glycolytic genes including hexokinase 2 (*HK2*), pyruvate dehydrogenase kinase 1 (*PDK1*), and lactate dehydrogenase A (*LDHA*). Additionally, MYC promotes glutamine uptake and mitochondrial glutaminase (GLS1) activity, which feeds into the tricarboxylic acid cycle and further contributes to lactate overproduction (19, 20).

However, cytogenetic studies did not reveal MYC gene rearrangement. This suggests alternative mechanisms of MYC upregulation, phenomena increasingly recognized in aggressive lymphomas without classic MYC translocations. MYC copy number gains and amplification have also been increasingly recognized as alternative mechanisms driving MYC protein overexpression in lymphomas lacking canonical translocations. Studies indicates that MYC amplification, even in the absence of chromosomal rearrangement, can lead to MYC protein overexpression and confer aggressive biological behavior and adverse prognosis comparable to that observed in MYC-rearranged cases (21, 22). Accordingly, the marked MYC protein expression observed in our patient may be related to MYC copy number alterations and/or amplification. However, due to tissue limitations, comprehensive FISH analysis evaluating MYC copy number status could not be performed in this case. Nevertheless, this observation underscores the importance of incorporating MYC copy number evaluation in future cases exhibiting aberrant MYC protein expression to fully characterized the underlying genetic mechanisms. Beyond genetic alterations, epigenetic dysregulation may represent a critical yet underappreciated mechanism driving MYC overexpression and metabolic transformation in FL. Aberrant DNA methylation patterns, histone modifications, and dysregulated chromatin remodeling complexes can collectively alter MYC transcriptional activity without requiring structural genetic changes (23). The identification of *ARID1A* mutation, a key component of the chromatin remodeling complex (24), further support the role of epigenetic dysregulation in driving aggressive phenotype in this case. The interplay between genetic mutations and epigenetic modifications likely created a permissive chromatin landscape enabling sustained MYC expression and metabolic derangement. Further studies incorporating methylation profiling and chromatin accessibility analysis may elucidate the specific epigenetic mechanisms underlying MYC-driven metabolic transformation in aggressive FL variants.

In this patient, we hypothesize that sustained MYC signaling drove an extreme form of metabolic reprogramming, resulting in relentless lactate accumulation. Several observations support this tumor cell-intrinsic mechanism. First, serum lactate levels fluctuated in parallel with tumor burden, rising during progression and transiently normalizing after effective cytoreduction. Second, lactate levels were markedly elevated relative to LDH, which remained near-normal initially, a pattern inconsistent with typical cellular breakdown but consistent with active glycolytic flux. Third, the patient had no diabetes, liver cirrhosis, tissue hypoxia, or medication exposure that could account for type A lactic acidosis. Fourth, serial biopsies consistently demonstrated strong MYC protein expression, suggesting persistent metabolic dysregulation.

The failure of different treatment regimens underscores the profound therapeutic challenge posed by this MYC-driven, metabolically dysregulated FL variant. Although direct MYC-targeted therapies remain elusive, several emerging strategies may address MYC-aberrant lymphomas. Intensified chemotherapy with targeted agents represents one approach. Dose-adjusted EPOCH-R combined with BET bromodomain or PI3K inhibitors, have shown preclinical efficacy in MYC-aberrant lymphomas (25, 26). Immune checkpoint modulation also holds promise. Tumor-derived lactate not only reflects heightened glycolytic flux but also reshapes the immune microenvironment, promoting M2 macrophage polarization and immune evasion via the CD47–SIRPα axis (27, 28). Novel agents targeting CD47, or bispecific antibodies combining CD47 blockade with CD20 targeting, have demonstrated preliminary efficacy in relapsed or refractory B-cell non-Hodgkin lymphoma (29). These approaches may counteract lactate mediated immunosuppression. For aggressive, chemotherapy refractory FL, CD19 directed chimeric antigen receptor (CAR) T-cell therapy represents a potentially curative option. However, the acidic, lactate-rich tumor microenvironment may impair CAR-T cell function, necessitating pre-conditioning with metabolic modulators to optimize therapeutic efficacy.

Finally, clear evidence of clonal evolution underscores the role of ongoing genetic diversification in therapeutic resistance (30). While no therapies directly target clonal dynamics, serial molecular monitoring to detect emerging subclones and their acquired vulnerabilities may be crucial. An adaptive, precision-based approach that integrates both genomic and metabolic insights may ultimately be required to address the biological complexity and treatment refractoriness observed in such aggressive FL cases.

## 4 Conclusion

This case highlights a rare, aggressive variant of FL grade 3A marked by recurrent severe type B lactic acidosis, persistent MYC overexpression, and sequential emergence of monoclonal immunoglobulins reflecting ongoing clonal evolution. The findings emphasize the importance of early recognition, metabolic monitoring, and consideration of novel therapeutic strategies targeting metabolic reprogramming and genomic vulnerabilities in similarly aggressive FL patients.

## Data Availability

The original contributions presented in the study are included in the article/supplementary material. Further inquiries can be directed to the corresponding author.
